# Investigating the structural network underlying brain-immune interactions using combined histopathology and neuroimaging: a critical review for its relevance in acute and long COVID-19

**DOI:** 10.3389/fpsyt.2024.1337888

**Published:** 2024-03-25

**Authors:** Zora Kikinis, Agustin Castañeyra-Perdomo, José Luis González-Mora, Richard Jarrett Rushmore, Poliana Hartung Toppa, Kayley Haggerty, George Papadimitriou, Yogesh Rathi, Marek Kubicki, Ron Kikinis, Carina Heller, Edward Yeterian, Bianca Besteher, Stefano Pallanti, Nikos Makris

**Affiliations:** ^1^ Department of Psychiatry, Psychiatry Neuroimaging Laboratory, Brigham and Women’s Hospital, Harvard Medical School, Boston, MA, United States; ^2^ Universidad de La Laguna, Área de Anatomía y Fisiología. Departamento de Ciencias Médicas Básicas, Facultad de Ciencias de la Salud, San Cristobal de la Laguna, Spain; ^3^ Universidad de La Laguna, Instituto Universitario de Neurosciencias, Facultad de Ciencias de la Salud, San Cristobal de la Laguna, Spain; ^4^ Department of Anatomy and Neurobiology, Boston University School of Medicine, San Cristobal de la Laguna, Spain; ^5^ Departments of Psychiatry and Neurology, Athinoula A. Martinos Center for Biomedical Imaging, Massachusetts General Hospital, Harvard Medical School, Boston, MA, United States; ^6^ Department of Radiology, Brigham and Women’s Hospital, Harvard Medical School, Boston, MA, United States; ^7^ Department of Psychiatry and Psychotherapy, Jena University Hospital, Jena, Germany; ^8^ Department of Psychology, Colby College, Waterville, ME, United States; ^9^ Department of Psychiatry and Behavioural Science, Albert Einstein College of Medicine, Bronx, NY, United States; ^10^ Istituto di Neuroscienze, Florence, Italy

**Keywords:** brain-immune interactions, inflammatory reflex, acute COVID-19, post-acute or long COVID-19, PASC, neuroinflammation, histopathology, neuroimaging

## Abstract

Current views on immunity support the idea that immunity extends beyond defense functions and is tightly intertwined with several other fields of biology such as virology, microbiology, physiology and ecology. It is also critical for our understanding of autoimmunity and cancer, two topics of great biological relevance and for critical public health considerations such as disease prevention and treatment. Central to this review, the immune system is known to interact intimately with the nervous system and has been recently hypothesized to be involved not only in autonomic and limbic bio-behaviors but also in cognitive function. Herein we review the structural architecture of the brain network involved in immune response. Furthermore, we elaborate upon the implications of inflammatory processes affecting brain-immune interactions as reported recently in pathological conditions due to SARS-Cov-2 virus infection, namely in acute and post-acute COVID-19. Moreover, we discuss how current neuroimaging techniques combined with *ad hoc* clinical autopsies and histopathological analyses could critically affect the validity of clinical translation in studies of human brain-immune interactions using neuroimaging. Advances in our understanding of brain-immune interactions are expected to translate into novel therapeutic avenues in a vast array of domains including cancer, autoimmune diseases or viral infections such as in acute and post-acute or Long COVID-19.

## 1 Introduction

Neuroscience as shaped in the early 1900s by such researchers as Ramon y Cajal, Golgi and Sherrington interacted with the field of immunology as early as the 1920s, and the two fields then developed along parallel paths (see e.g., 1). It was not until the 1950s that neuroscience and immunology began to relate more deeply when the notion arose that chemical substances such as hormones or other soluble mediators for balancing homeostasis possessed activities that could be placed into both the immune and neural categories. Subsequently, breakthroughs in experimental animal research in the 1990s (e.g., 2–6) provided evidence for direct immune-brain interactions and the formulation of the neuroinflammation reflex (7). These developments were a result of the integration of biochemistry, physiology and medicine after the early 1960s and are eloquent and successful examples of the flourishing “modern neuroscience” paradigm we are currently in (e.g., 8). Importantly, the neuroinflammation reflex is the embodiment of interactions between the immune and nervous systems and constitutes the basis of the neuroimmune network, through which the nervous system modulates the immune system and vice versa. The neuroimmune network involves several brain structures including cerebral cortical regions such as the prefrontal cortex, limbic and paralimbic regions (e.g., cingulate cortex and insula), and autonomic system structures including the hypothalamus and brainstem (e.g., 9–12). These gray matter structures are interconnected via fiber pathways, comprising brain circuits that operate as a unified network. We refer in this review to this set of brain structures as the “neuroimmune network.” By modulating immunological homeostasis and immune responses, these neuronal circuits are of critical relevance for human survival, and their failure leads to disease (1). Through the neuroimmune network, inflammatory processes could be modulated in real time and in a reflex-like fashion, a notion of great importance in such pathological conditions as acute and post-acute COVID-19. The pro-inflammatory cytokine release syndrome (cytokine storm) in severe COVID-19 is a surge of enormous inflammation that results in high mortality (e.g., 13–16). Histopathological evidence indicates that SARS-CoV-2 infection produces a dysfunction of the vascular endothelium including oxidative stress and inflammation (e.g., 17, 18). Given that the endothelium is necessary for the maintenance of tissue homeostasis throughout the body, endotheliopathy in COVID-19 results in multi-organ injury (e.g., 18–22). This immune/inflammatory response in acute COVID-19 can shift to post-acute chronic COVID-19 (PASC), which is also called Long COVID, a neurological condition characterized by neuroinflammation, the pathophysiology of which is not well understood (e.g., 12). In recent years, neuroinflammation has been investigated using neuroimaging techniques such as PET (e.g., 12, 23–25) in COVID-19 studies, which have shown structural and functional alterations in several parts of the brain and in the brainstem in particular (e.g., 10). Furthermore, diffusion MRI enables us to investigate neuroinflammation in the brain white matter (e.g., 26–29). Thus, the neuroimmune network can be investigated in its entirety, i.e., the gray matter brain centers and the interconnecting axonal fiber pathways, using neuroimaging in clinical conditions. Neuroimaging provides unique insight in detecting, localizing and characterizing brain lesions *in vivo*. Nevertheless, brain autopsy followed by histopathological examination still remains the undisputable method for confirming and understanding disease. In this paper we review recent developments regarding brain-immune interactions, the anatomy of the neuroimmune network as affected in acute COVID-19 and Long COVID, and, finally, how the neuroimmune network can be investigated using combined histopathology and neuroimaging in these clinical conditions.

## 2 Relevant sections

### 2.1 Overview of COVID-19 following SARS-CoV-2 virus infection

Viral replication, immune hyperactivation and post-acute sequelae or Long Covid is a multi-phasic stage characterization currently used for COVID-19 following SARS-CoV-2 virus infection (30). According to this three-stage view, SARS-CoV-2 virus enters the host body and replicates itself. Following an incubation period of approximately five days, a clinical phenomenology of an upper respiratory system infection, fever, muscle fatigue and pain appears. The organism initially reacts with innate and adaptive immune responses and, in non-severe COVID-19, symptoms are commonly resolved within a four-week period (e.g., 30). Conversely, in severe COVID-19, SARS-CoV-2 virus can escape immunity and eventually a second phase of immune hyperactivation can take place. This second phase has been associated principally with an abnormal response of immune cells of the host such as macrophages and natural killer cells, which can function abnormally and promote a dysregulated release of interferons and proinflammatory cytokines such as IL-1b, IL-6 and IL-12 resulting in PAN-optosis and eventually in hypercytokinemia or cytokine storm (e.g., 30–33). This sequence of events usually leads to overwhelming systemic inflammation and multiorgan failure manifested as stroke, lung injury, cardiac, liver and kidney injuries as well as vasculopathy, secondary infections and sepsis with high mortality (30, 32, 34, 35). Although in COVID-19 survivors, symptoms would usually resolve within one to four weeks from their initial appearance, a number of patients would continue reporting symptoms, such as fatigue, post-exertional malaise, headache, dyspnea/shortness of breath, anosmia and cognitive dysfunction beyond this period of time (36). If these symptoms persist “for at least two months occurring within three months after COVID-19 infection which cannot be explained by an alternative diagnosis” the World Health Association (WHO) has termed this clinical condition as post-COVID-19 condition (PCC), which is synonymous with “Long COVID” or Post-Acute Sequelae of COVID-19 (PASC) (34, 37). Histopathologically, it seems that SARS-CoV-2 virus infection produces an endotheliopathy and that the pulmonary capillary endothelium is the most common entry in the body for viral replication and, eventually, for the virus to get access in the blood stream (35, 38–40). Endothelial damage is diffuse and extends beyond the respiratory system impairment underlying such multi-system, multi-organ clinical manifestations of COVID-19 (19, 20, 22) as those present in the cardiovascular system, the liver, the kidney and the brain (e.g., 21, 35).

### 2.2 Neurohistopathology and neurology in acute COVID-19 and Long COVID following SARS-Cov-2 virus *infection*

**Figure 1:**
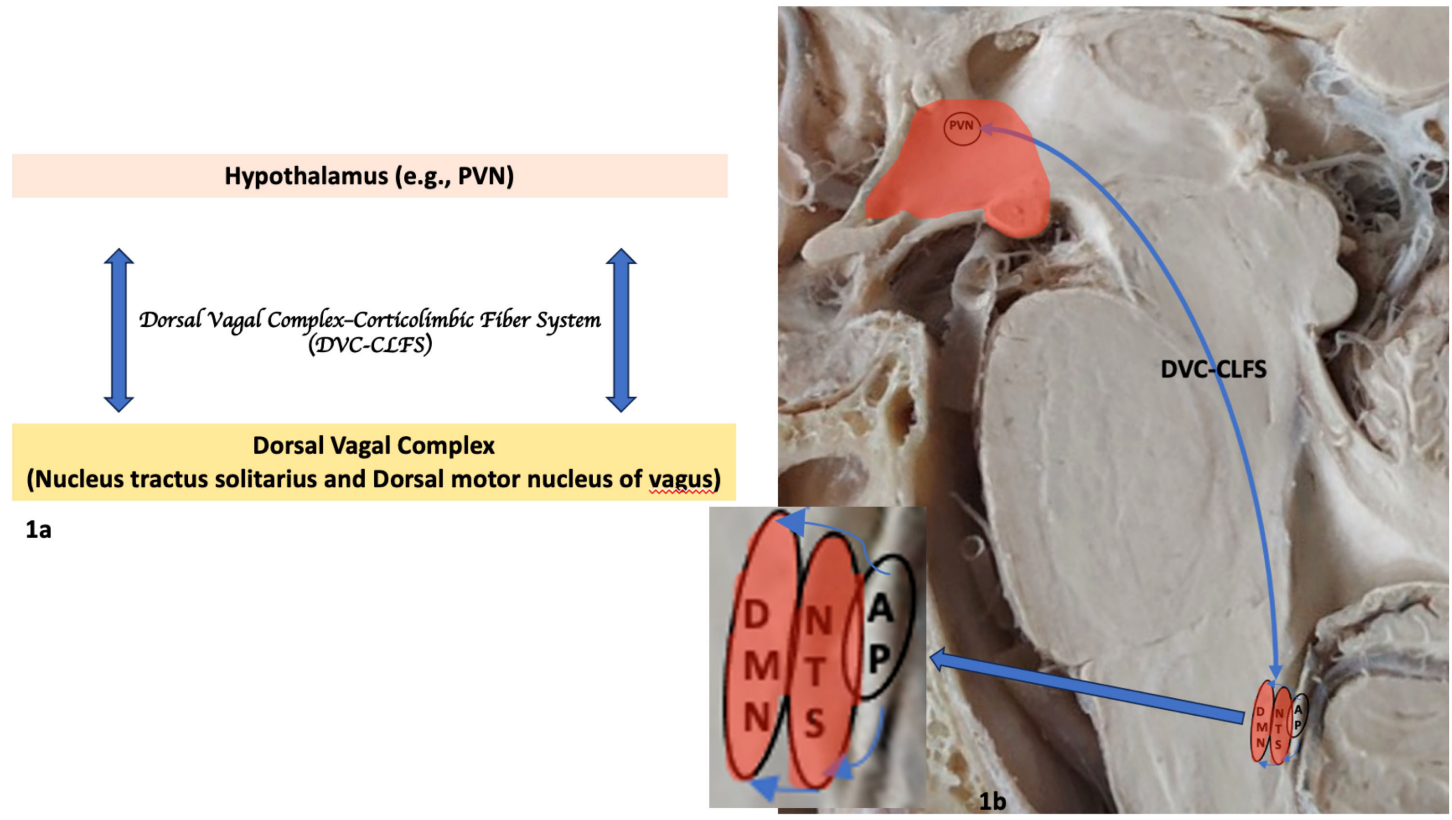
Principal gray matter structures and white matter fiber pathways of the human neuroimmune network involving the brainstem and hypothalamus. **(A)** Diagrammatic representation of the neuroimmune network depicting structures in the CNS. **(B)** Anatomical and histological representation of neuroimmune network’s principal structures in the brainstem and ventral diencephalon. The magnified box in 1b indicated by the arrow shows the dorsal vagal complex or DVC (NTS and DMN of vagus) connectivity and its relationship with the area postrema (AP) in further detail. AP, area postrema; DMN, dorsal motor nucleus of vagus; DVC-CLFS, Dorsal vagal complex-corticolimbic fiber system; NTS, nucleus of the solitary tract; PVN, paraventricular nucleus of hypothalamus.

**Figure 2:**
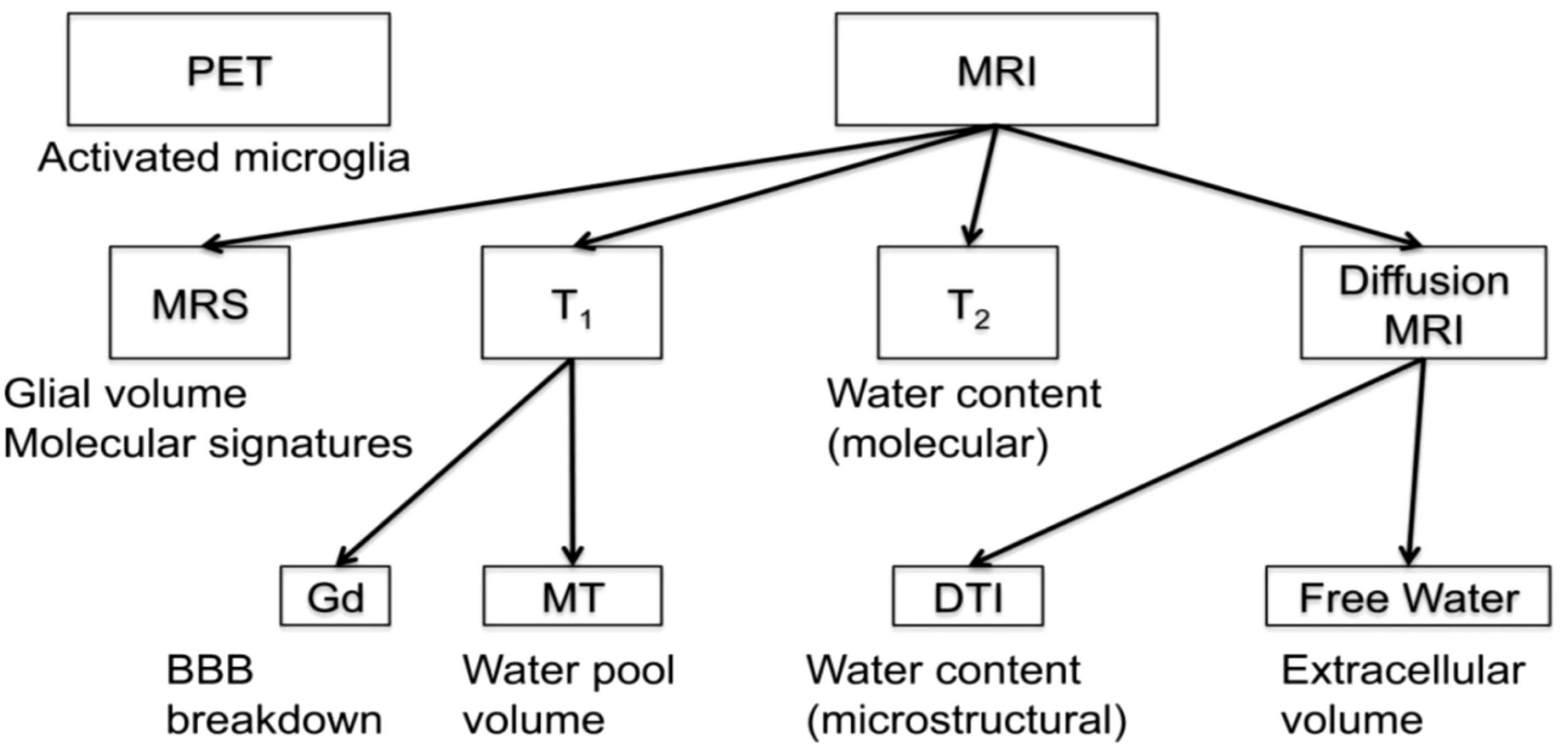
Array of imaging methods for the study of neuroinflammation (by permission from 29). BBB, blood brain barrier; DTI, diffusion tensor imaging; Gd, gadolinium; MRI, magnetic resonance imaging; MRS, magnetic resonance spectroscopy; MT, magnetization transfer; PET, positron emission tomography; T1, T1-weighted MRI; T2, T2-weighted MRI.

**Figure 3:**
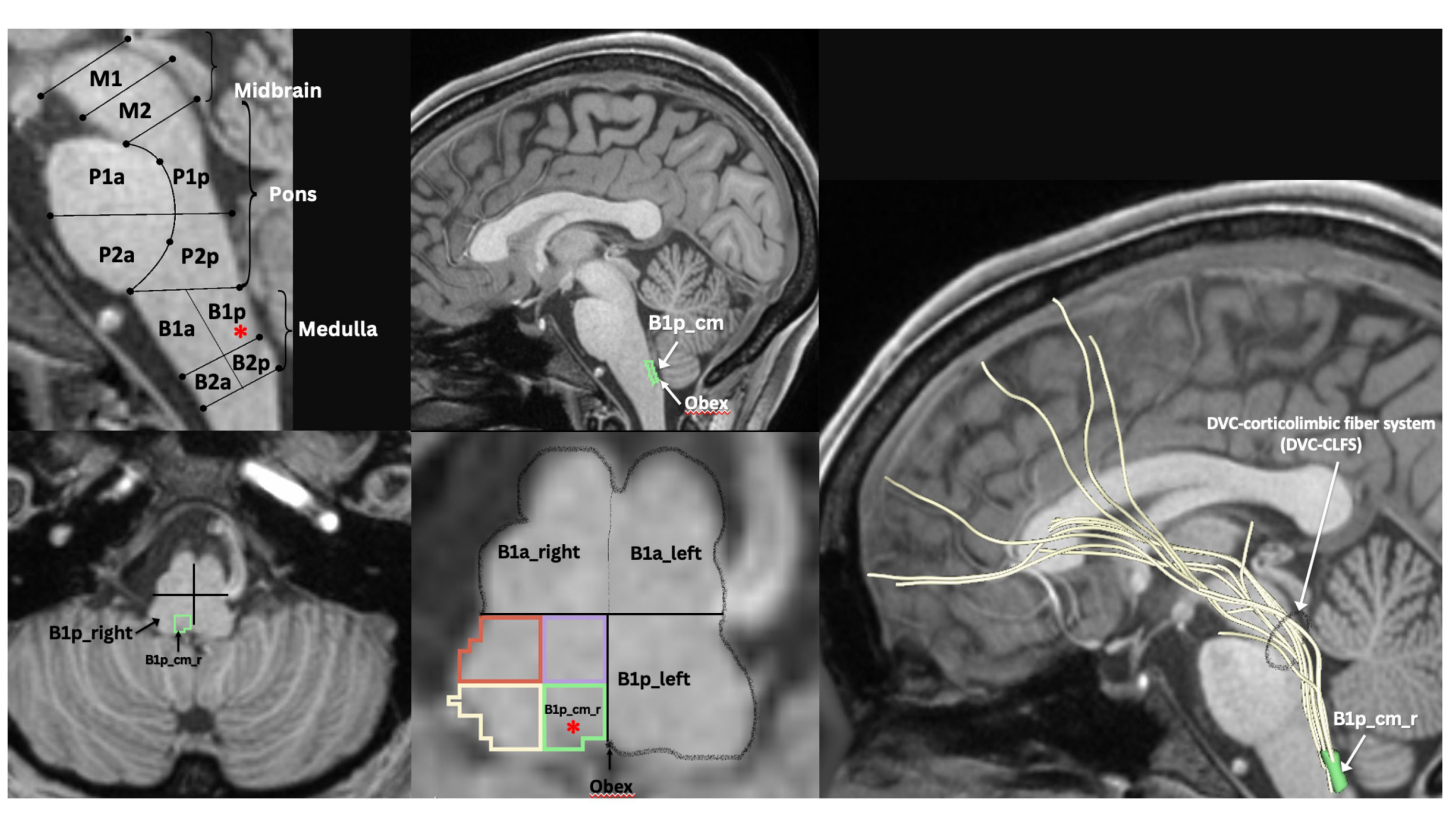
MRI-based morphometric brainstem methodology for ROI anatomical definition and fiber tract delineation: Dorsal vagal complex ROI, i.e., B1p_cm (i.e., caudal-medial quadrant of posterior upper medulla, indicated by red asterisk in sagittal and axial views), sampling and white matter fiber reconstruction using dMRI tractography methods as previously described by our group (47–52).

Neurological complications of acute COVID-19 involve the brain, cranial nerves and peripheral nerves (41) with clinical manifestations that include thromboembolic strokes, intracranial hemorrhages as well as encephalitis, meningoencephalitis and neuropathy. It is not uncommon that acute neurological phenomenolgy may persist for weeks and also for a more prolonged period, which can last from months to years after recovery from the initial infection (42). Mechanistically, the neuropathogenesis of acute COVID-19 remains unclear (41, 43), and the elucidation of whether the neurological effects in COVID-19 are a) mediated directly by SARS-CoV-2 virus or b) an indirect effect of the virus itself (namely, hypercoagulopathy) or an immune-mediated/autoimmune-mediated (such as the cytokine storm) neuroinflammation (41) is a matter of great importance biologically and clinically. Neurohistopathological studies have demonstrated acute hypoxic ischemic injury in the cerebrum and cerebellum in brain tissue of patients who have undergone autopsy (e.g., 44–46). More specifically, Solomon and colleagues (2020) reported that microscopic examination has shown neuronal loss in the frontal lobe, hippocampus, and Purkinje cell layer of the cerebellum. Another study on a post-mortem case series by Matschke and colleagues (2020), showed pronounced neuroinflammatory alterations in the brainstem such as in the upper medulla oblongata (44 with microscopic details in **Figures 1**–**3**). Schurinck and colleagues (2020) by contrast, in a prospective autopsy study investigating a cohort of 21 patients with lethal COVID-19, showed “a severe innate inflammatory state” with massive activation of microglia in the brain. Based on their findings and in light of other histopathological studies reporting the presence of SARS-CoV-2 infected cells in the brain (46, 53), they suggested that a large inflammatory response may lead swiftly to viral clearance “shifting the pathology towards an autonomous immune-mediated reaction.” Schurinck et al. also emphasized the extensive presence of inflammation in the medulla oblongata, which is critically important in regulatory respiratory functions, which could well contribute to the respiratory failure occurring in these patients (45). Thus, it seems likely that while direct viral effects cannot be easily estimated in acute COVID-19, the clinical profile of brain inflammation is more likely produced by immune-mediated responses or autoimmune reactions (54). Furthermore, the intense virus infection-related systemic inflammatory response can lead to breakdown of the blood-brain barrier (BBB), which could allow entry into the central nervous system (CNS) of peripheral inflammatory molecules such as cytokines producing autoimmune encephalitis (55, 56). Besides short-term or acute consequences, there are also important long-term or post-acute consequences of COVID-19 that also go beyond the respiratory system and constitute the post-acute sequelae of the Long COVID condition. Long COVID occurs in at least 10% of patients, which had severe acute respiratory syndrome coronavirus 2 (SARS-CoV-2) infection (57). By 2022, more than 65 million individuals worldwide are estimated to have long COVID, a number that is currently rising (58). This illness is multisystemic affecting seriously the nervous system with such new onset manifestations as myalgic encephalomyelitis/chronic fatigue syndrome (ME/CFS) and dysautonomia (especially postural orthostatic tachycardia syndrome (POTS) (59, 60), which can become lifelong conditions (61). While the picture of Long COVID’s causes remains unclear, several hypotheses have been suggested regarding its pathogenesis. As reviewed recently by Davis and colleagues (2023) these mechanisms include “immune dysregulation with or without reactivation of underlying pathogens (such as Epstein–Barr virus and human herpesvirus-6), microbiota and virome disruption (including SARS-CoV-2 persistence), autoimmunity and primed immune cells from molecular mimicry, microvascular blood clotting with endothelial dysfunction, and dysfunctional neurological signaling in the brainstem and/or the vagus nerve” (modified from **Figure 3** in Davis, 57). Long COVID is characterized by a plethora of symptoms and bears remarkable similarities to such viral-onset illnesses as ME/CFS and POTS (57). More specifically, frequent neurological symptoms associated with Long COVID are cognitive impairment (i.e., brain fog), memory loss, fatigue, unfreshening sleep, pain and post-exertional malaise (11). A principal condition underlying these behaviors is neuroinflammation, the cause of which may be a chronic or relapsing neuroinflammatory process initiated by initial SARS-CoV-2 virus infection, which can lead to increased permeability of the blood-brain barrier (11, 12, 57). Neuroinflammation seems to be widespread (25, 62) and involves important nervous centers and fiber pathways that play a key role in several biobehavioral functions. Given the importance of endotheliopathy and BBB dysfunction in understanding similarities and differences in pathological mechanisms in acute and Long COVID-19 and in other neurodegenerative diseases, as well as ways in which these disorders may be interrelated (e.g., comorbidity), we will elaborate further in this regard in section 3. We will review neurologically based functions in more detail in sections 4 and 5, addressing autonomic, neuroendocrine, affective or limbic, and cognitive aspects. Furthermore, we will emphasize the role of the known neuroanatomical structures underlying brain-immune interactions, namely the neuroimmune network in the CNS in sections 4 and 5. Finally, in section 6 how we will review how to investigate the neuroimmune circuitry using current neuroimaging.

### 2.3 Endotheliopathy, BBB dysfunction, and their association with neuronal injury in acute and long COVID-19

Endotheliopathy and BBB dysfunction are considered key underlying pathophysiological processes in acute and Long COVID-19 (63). Endotheliopathy has been reported to result from SARS-CoV-2 protease activity, whereas complement activation can be involved in hypoxic neuronal injury and blood–brain barrier (BBB) dysfunction (63, 64). The BBB is a complex structure comprising endothelial cells that form tight junctions to protect the neural tissue. These cells impede the entry of different substances, cells or molecules that, if penetrating the BBB, would induce CNS pathology. The permeability of the BBB is regulated by various perivascular structures such as microglia, pericytes, astrocytes and the basement membrane, which form a physical barrier (see e.g., 11, 65). At a mature stage, the BBB is stabilized by highly specialized perivascular structures. BBB restriction of paracellular and transcellular transport of solutes is affected by a number of processes, as detailed by Zhao and colleagues (66). BBB permeability is altered following the addition of extracted SARS-CoV-2 spike protein, resulting most likely from a pro-inflammatory response producing endothelial damage (63). In a recent study of the pathophysiologic mechanism of encephalopathy and prolonged comatose or stuporous state in severally ill patients with COVID-19, Dalakas and colleagues (67) indicated that a) high levels of circulating proinflammatory cytokines due to SARS-CoV-2 infection possibly disrupt the BBB allowing antibodies and other inflammatory mediators to enter the brain parenchyma, as recently shown by other investigators (68); b) systemic effects related to multiorgan failure may be additional factors facilitating BBB disturbance; and c) endothelial cells, which are a structural part of the BBB, can also be directly affected by the virus or the circulating cytokines resulting in endotheliaitis, which further compromises BBB integrity. Thus, as concluded by Dalakas and colleagues “the cause and consequence of disturbed BBB needs to be elucidated” (67). The effects of endotheliopathy and BBB leakage can be quite serious, given their contribution in acute and/or chronic neural inflammation and neuronal injury as well as associated neurological clinical manifestations (63). It should be pointed out that breakdown of the BBB can also be present in clinical conditions other than COVID-19, including acute disorders such as traumatic brain injury, stroke, and seizures or neurodegenerative diseases such as Alzheimer’s disease (AD), vascular dementia, small vessel disease and multiple sclerosis (MS) (66, 69, 70). Importantly, we are able to detect BBB leakage using neuroimaging. The most commonly applied technique is a *T*1-weighted dynamic contrast-enhanced (DCE)-MRI, during which a gadolinium-based contrast agent is injected intravenously; leakage of the contrast agent can be detected when it crosses the damaged BBB (e.g., 70, 71). It should be noted that there is substantial comorbidity between COVID-19 and AD (72, 73) and the chronic neurological sequelae in Long COVID are also present in AD clinical phenomenology. Furthermore, there is evidence indicating *APOE* e4e4 genotype as a potential genetic determinant for the development of severe COVID-19 (74), and *ApoE* e4e4 homozygotes are more likely to be COVID-19 test positive (75). Furthermore, *ApoE* e4e4 homozygotes were found to be at the highest risk of sporadic AD (73, 76). However, the mechanism underlying this association remains unclear (74). Another lesson learned by studying neurodegeneration in MS, AD and Parkinson’s disease (PD) that may bear insight in approaching Long COVID and myalgic encephalomyelitis/chronic fatigue syndrome (ME/CFS) is the relevance of assessing in the cerebrospinal fluid (CSF) inflammatory biomarkers (11) or other potential biomarkers for COVID-19 such as tau (77). This may also be important information in reasoning on diagnosis and prognosis as well as such aspects as disease activity and response to treatment (11). Moreover, the relationship between COVID-19 and tau pathology remains an unsolved problem and topic of ongoing research. Although in experiments using brain organoids, SARS-CoV-2 virus affects neurons showing altered distribution of tau throughout the neurons from axons to soma, hyper-phosphorylation and neuronal death, it remains unclear whether these effects are produced directly by the virus or other factors such as neuronal stress (78). Furthermore, it has been shown in experimental animals that pathogenic tau can “seed” synaptically connected cells with further spreading of the disease (79, 80). This process is carried out by extracellular micro-vesicles (EVs) or “exosomes”. Autopsy studies have shown tau deposits in brain tissue of COVID-19 patients (77). In addition, EVs containing tau are also present in cerebrospinal fluid and plasma of patients with mild AD and frontotemporal dementia (FTD) (77, 81). Nevertheless, regarding the relationship between the two clinical conditions, as reported by Wang and colleagues, “whether COVID-19 might trigger new-onset Alzheimer’s disease or accelerate its emergence is unclear” (82). That said, a highly important topic in any clinical condition including viral infections and neurodegenerative disorders is how the brain and the immune system are interrelated.

### 2.4 Brain-immune interactions

It was not until breakthroughs in experimental animal research in the 1990s (e.g., 2–4, 6) provided evidence of direct immune-brain interactions that the immune and nervous systems would be considered strongly and dynamically related (83). A critical discovery in this regard has been the demonstration of the cholinergic anti-inflammatory pathway as a means of direct neural modulation in suppressing inflammation by the motor branch of the vagus nerve (84, 85), as well as the conceptualization and demonstration of the neuroinflammation reflex as a reflexive hard-wired selective neural pathway with inflammation-sensing and inflammation-suppressing functions (7). Since then, the topic of nervous system (NS) modulation of immunity has been a highly active area of research and has provided considerable insights into the anatomical and molecular mechanisms underlying the brain-immune dynamics during inflammation. Modulation of inflammation by the NS appears to be done by way of neural and humoral pathways. In COVID-19, the pathological process is commenced by initial SARS-CoV-2 virus infection. The NS involvement seems to be triggered either indirectly by the inflammatory peripheral response in the body or directly by the virus per se or any inflammatory molecules that gain access to the CSF due to increased BBB permeability. According to this model, the afferent component of the vagus nerve, which innervates various viscera such as the airways, lungs, heart, liver, spleen, stomach and intestines, is stimulated by pro-inflammatory cytokines produced by the peripheral inflammatory process in SARS-CoV-2 virus infection (7, 86, 87). Peripheral inflammatory status information is thus conveyed to the nucleus of the solitary tract (NTS) in the medulla. The connections of the NTS are numerous and complex, nevertheless its connection with the dorsal motor nucleus of the vagus (DMN) seems to be one of the most relevant in the brain for immune system interactions in response to inflammation. The NTS-DMN ensemble (or dorsal vagal complex (DVC)) seems to be where the coordination of vagal afferent and efferent responses takes place (7, 88). Subsequently, through efferent outflow, the vagus inhibits activated tissue macrophages in releasing pro-inflammatory cytokines. Thus, through this neural pathway, called the “cholinergic anti-inflammatory pathway” (84, 85), the vagus is able to suppress inflammation in real time (7, 88). The afferent and efferent branches of the vagus together with the NTS and DMN constitute the neuroanatomical substrate of the ‘inflammatory reflex’, a hard-wired neural circuit that aims to inhibit acute inflammation in real time. The discovery of the inflammatory reflex has been a fundamental stepping-stone in the understanding of brain-immune interactions; nevertheless, the complete neural circuitry involved in immune modulation remains to be identified with clarity. Although it is conceivable that circuitries including the DVC should be part of a larger distributed neuroimmune network, the exact definition of a comprehensive neuroimmune system beyond the inflammatory reflex that modulates the immune system is still not well understood. This is due, in part, to the fact that the autonomic nervous system (ANS) is arguably one of the most enigmatic aspects of the NS anatomically and functionally. Consistent with this view, the neuroimmune network would involve centers and fiber pathway connections across the CNS, encompassing the cerebral cortex, subcortical forebrain structures, the cerebellum and the brainstem. Following the overall anatomical organization of the ANS structural architecture (89), in the efferent or motor path of the system, there should likely be a hierarchical process with the involvement of antecedent neurons to premotor and motor neurons innervating preganglionic neurons, which in turn act upon postganglionic neurons to affect the target organs. In addition to the structural aspect of the neuroimmune network, there is also the humoral side that has to be equally considered. Furthermore, the pain system plays a role in this schema as well (7). Realizing the complexity of a comprehensive neuroimmune network, in which a multitude of structures and circulating substances are involved, we can appreciate that our current knowledge is scant and that any attempt to specify a precise and complete blueprint of the neuroimmune network would lack clarity. Nevertheless, in an effort to schematize the neuroimmune network, we could attempt an approach based on consolidated basic principles and known facts, namely the inflammatory reflex and the organizational architecture of the ANS. Thus, at a first approximation, the NTS and the DMN of the vagus, and their direct connections supplied principally by fiber pathways such as the medial forebrain bundle (MFB) and the dorsal longitudinal fasciculus of Schutz (DLF) with the cortico-limbic system, seem to be implicated in the structural brain-immune connectome. We must keep in mind that this is a simplistic and hypothetical view of the neuroimmune network blueprint and that humoral and pain processing pathways seem to be involved as well. Based on available literature and neuroanatomical knowledge of the ANS (e.g., 89, 90), we illustrate in **Figure 1A** simplified blueprint of neuroimmune network anatomy involving the NTS, the DMN of the vagus and the hypothalamus, in particular the paraventricular nucleus (PVN), as well as the white matter fiber system that interconnects these structures. Moreover, the humoral pathway involving the adrenocorticotropic hormone (ACTH) and the hypothalamic-pituitary-adrenal (HPA) axis associated with stress and pain has been considered an integral part of the neuroimmune network given the adrenergic and noradrenergic inhibitory effect on macrophage activation resulting in suppression of the synthesis of tumor necrosis factor (TNF) and other cytokines, which leads to an anti-inflammatory effect (7, 86, 87, 91–93).

### 2.5 How can neuroinflammation or direct CNS injury affect the immune response?

The clinical phenomenology of COVID-19 and its schematic characterization as a multi-phase sequence of events, namely viral replication, immune hyperactivation and Long COVID (e.g., 30), is useful for a clearer understanding of the pathophysiological process of the disease as well treatment approaches. More specifically, as Sapir and colleagues (2022) denoted and summarized, the initial viral invasion and replication produces an upper respiratory tract infection, fever, muscle fatigue, pain and, given the viral active presence, antiviral agents can be used to decrease viral load, transmission, and prevent disease progress. In the phase of immune hyperactivation, pneumonia, vasculopathy, and multiorgan pathologies such as acute cardiac and renal damage, thromboembolic strokes, sepsis and secondary infections can occur. Given the systemic nature of the disease, monoclonal antibodies, anti-coagulants, immunosuppressants, oxygen, and antiviral drugs need to be used for treatment. Finally, in Long COVID where fatigue, headache, dyspnea, and anosmia are the common symptoms, a therapeutic approach using immunosuppressants and convalescent plasma therapy may be warranted. Histopathologically, a diffuse endotheliopathy is produced by the virus with consequent multi-organ injury, which can also affect the brain with several neuropathological alterations that we reviewed previously. Herein we focus on neuroinflammation, which can occur in the acute as well as the post-acute chronic phase. There are several hypotheses on the mechanisms by which neuroinflammation arises and establishes itself, although several aspects of these processes remain to be clarified (e.g., 94, 95). It has been hypothesized that the SARS-CoV-2 virus infection-related systemic inflammatory response can lead to increased permeability of the blood-brain barrier (BBB), through which peripheral inflammatory molecules such as cytokines can access the central nervous system to produce neuroinflammation (55, 56). Furthermore, the virus could have direct access to and effect on the CNS through such circumventricular organs (CVOs) as the area postrema (AP), the subforniceal organ (SFO) and the organum vasculosum of the lamina terminalis (OVLT), which are characterized by high permeability in the BBB and fenestrated capillaries and thus are easily reachable by the SARS-CoV-2 virus via the bloodstream (e.g., (96, 97). Although, to our knowledge, there is currently no published evidence supporting this idea, it is conceivable that the SARS-CoV-2 virus could enter the CNS through the AP, the SFO and the OVLT. One of the reasons for studying the CVOs in COVID-19 is that the AP, SFO, OVLT and PVN show high expression of angiotensin-converting enzyme 2 (ACE2), which is a binding site of entry in the cells for SARS-CoV-2 virus (e.g., 96, 97). In addition, the AP is connected with the NTS, whereas the SFO, OVLT and PVN form a pathway leading to the secretion of vasopressin or antidiuretic hormone (ADH), given that circulating angiotensin II (Ang II) readily reaches the SFO and the OVLT (98, 99). Ang II in turn stimulates the PVN via direct axonal connections to secrete ADH (e.g., 100, 101). In another pathway critical for central autonomic regulation, the AP is involved via its structural connectivity with the nucleus of the solitary tract (NTS), the dorsal motor nucleus of the vagus (DMN) as well as with the nucleus ambiguus, the rostral ventrolateral medulla and the PVN (e.g., 89, 102–104). Thus, both pathways involving the AP, SFO, OVLT and PVN are important in arterial blood regulation and cardiac function; consequently, their dysfunction could well lead to cardiovascular disease (e.g., 104–108). Furthermore, the AP affects the dorsal vagal complex (DVC), which is the basis of the inflammation reflex circuitry (7). Reflecting on these considerations with histopathological observations from brain autopsies of COVID-19 victims in our mind, we believe that there is a dire paucity of specific histopathological information targeting critical brain structures associated with the neuroimmune network as described herein. We believe it is crucial to accrue this essential information, especially in Long COVID where neuroinflammation seems to be an established chronic condition, and clarify whether the fundamental member structures of the neuroimmune network, in particular the NTS, DMN of the vagus and PVN are actually affected by an inflammatory process. Nevertheless, it already has been hypothesized that neuroinflammation affects the paraventricular nucleus of the hypothalamus, which leads to an array of biobehavioral disturbances associated with a chronic alteration of the HPA axis (11). The latter question could be addressed using current neuroimaging.

### 2.6 Investigating neuroinflammation in acute and long COVID-19 with neuroimaging

Histopathologically, neuroinflammation is characterized by the activation of the astrocytes and microglia, which represent the brain’s innate immune system (29, 109). For instance, pro-inflammatory cytokines that reach the brain through a damaged BBB can activate microglia, which in turn produces cytokines that initiate a cascade of events leading to a neuroinflammatory response. When this response is short-term, it may be beneficial and contribute to tissue repair; however, if it is prolonged, it may produce damage to the neighboring tissue. More specifically, a long-term neuroinflammatory response, lasting for weeks to years, could destroy the cellular and extracellular matrix of the brain tissue, such as neurons and oligodendrocytes including the axonal myelin sheaths. Ultimately, long-term neuroinflammation would lead to brain tissue atrophy and neurodegeneration, and consequently to damage of the brain circuitry in its entirety (28, 29, 110–114). A key to understanding the involvement of neuroinflammation with respect to the neuroimmune circuitry is our ability to localize and to monitor neuroinflammation with respect to where and when it begins and to maintain a record of these parameters as the neuroinflammatory process progresses. However, monitoring neuroinflammation *in vivo* in the brain using CSF markers is challenging and does not provide us with specific information on its location and extent in gray matter structures and fiber pathways (29, 115). Importantly, current neuroimaging provides us with a number of techniques that are demonstrated to be a valuable aid in addressing and, eventually, solving this problem. It should be pointed out that the technological advances in imaging during the past four decades have generated tremendous innovation in medicine. Indeed, MRI was rated by general practitioners in the USA as the principal contributor to medical practice in “the decade of the brain” (i.e., 1991–2000) (116). Neuroimaging caused a revolution in neurology and neurosurgery for diagnosis and therapeutic strategies, and it is an undisputed fact that clinical neurosciences and neuroimaging are mutually benefiting from a thriving symbiosis (117). A critically important factor underlying the applicability of imaging technology is that these techniques can be performed non-invasively and *in vivo*, and thus in clinical settings. Current neuroimaging approaches can contribute substantially in studying the neuroimmune network in normality and pathological conditions such as neuroinflammation. Brain structure analysis is commonly done using such imaging modalities as anatomical and quantitative magnetic resonance imaging (MRI) including different types of morphometric analysis by T1/T2-weighted MRI, structural connectivity using diffusion MRI (dMRI) tractography, and neurochemically using magnetic resonance spectroscopy (MRS). In current neuroimaging clinical research, brain circuits are usually studied using structural and functional connectivity imaging techniques, namely a combination of T1/T2-weighted MRI, diffusion MRI tractography and functional MRI connectivity. Using a combination of imaging techniques enables the performance of various complex studies, which can be structural and functional or of other nature such as metabolic and vascular (**Figure 2**). T1/T2-weighted MRI is optimal for gray matter analyses, whereas dMRI is used for white matter analysis and fiber tract delineation (e.g., 29). Functional analyses, by contrast, are performed using fMRI and functional connectivity analysis (fCONN), whereas metabolic studies are carried out using positron emission tomography (PET) (e.g., 29). Importantly, these neuroimaging techniques can also detect neuroinflammation, and to this end PET is the technique of preference (e.g., 29). PET enables us to detect neuroinflammation by using tracers such as Isoquinoline ligand 1-[2-chlorophenyl]-N-methyl-N-[1-methyl-propyl]-3-isoquinoline carboxamide (PK11195) (118), a ligand specific for the translocator protein-18-kd (TSPO) of the mitochondrial membrane (119). TSPO is highly expressed in activated microglia and to a lesser extent in reactive astrocytes and is thus detectable by PET in inflamed neural tissue (29, 120). With respect to T1-weighted imaging, there are several variants that can be of relevance in studying the neuroimmune network. T1-weighted imaging is a MRI technique used for anatomical characterization of gray matter brain structures and for gross delineation of white matter as well. Thus, T1-MRI is commonly used for MRI-based morphometric analysis (see e.g., 29, 121–123). With respect to neuroinflammation, there are two T1-weighted imaging techniques, namely gadolinium-enhanced T1, and magnetization transfer (MT) useful for the identification of neuroinflammation (28). By contrast, T2-weighted imaging is routinely used for edema detection, which appears hyperintense as compared to healthy brain tissue (124) because water has higher T2 than brain tissue and water content is increased in brain regions affected by an inflammatory process (125, 126). Fluid attenuation inversion recovery (FLAIR) and quantitative T2 can further highlight such hyperintensities, thus these techniques are also useful for the study of neuroinflammation (28, 127). Furthermore, because of edematous excessive water production in neuroinflammation, geometrical changes occur in the extracellular space of the affected area and are detectable by diffusion imaging. Thus dMRI can be used as an indirect method to measure neuroinflammation (27, 128, 129). Importantly, using a particular dMRI model called the “free-water model,” we are able to detect directly the specific contribution of extracellular water that is “free” or far away from brain tissue membranes (129). Finally, MRS allows us to detect specific metabolites, such as N-acetyl-aspartate (NAA), myo-inostitol (MI), choline compounds (Cho) and total creatine (tCr), some of which are involved in neuroinflammation (130–132). The data obtained by these techniques *in vivo* allow us to observe anatomical-clinical and other types of correlations, which provide us with valuable and unique information that cannot otherwise be obtained clinically, i.e., by traditional anatomical, histopathological, physiological or metabolic tests. Specifically, in investigations of neuroimmune network neuroinflammation, we would need to obtain an array of data that provide us with information regarding the neuroanatomy of the component structures of this circuitry as well as the structural, physiological and pathological status of these tissues. In essence, it is the “what, where, which and how” questions we need to answer, namely “what” is the anatomy, i.e., the individual structures of the circuitry we are investigating including the degree or extent of the pathology involving these structures; “where” is this circuitry in the individual brain under investigation; “which” is the pathological process affecting this circuitry, and “how” the mechanistic aspect of the pathological process affects this circuitry. Multimodal neuroimaging is an important instrument to address these key questions. Herein we illustrate a multimodal neuroimaging approach combining T1- weighted MRI morphometric analysis techniques and dMRI tractography (50, 51) to reconstruct the gray and white matter structures of the neuroimmune network in normal and clinical datasets as described in previous reports of our group (47, 49, 52, 133) (**Figure 3**).

**Figure 4:**
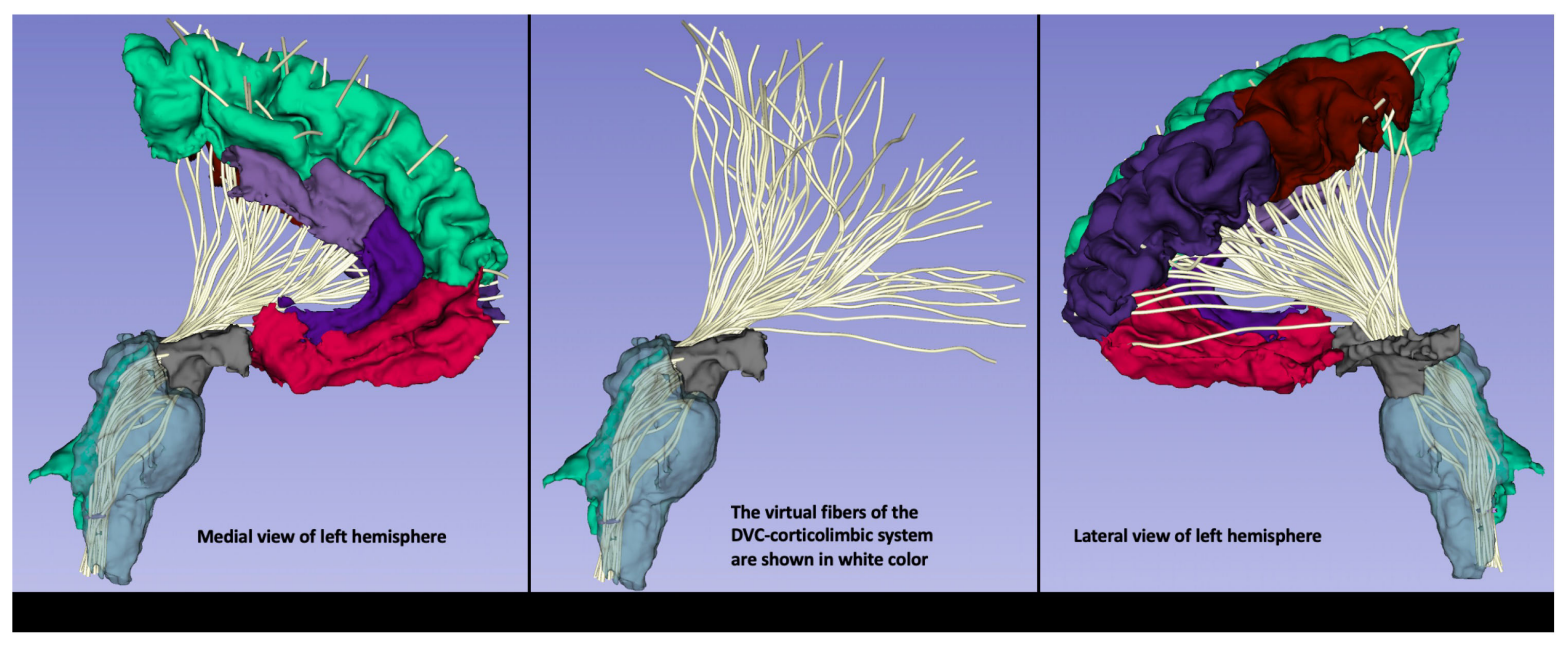
An exemplar illustration of the DVC-corticolimbic fiber system (DVC-CLFS) in a subject affected by post-acute COVID-19 (PASC) or Long COVID. The cortico-limbic structural connectivity of the dorsal vagal complex (DVC) ROI is illustrated as reconstructed using MRI-based anatomical cortical parcellation for the cortex of the frontal lobe and subcortical segmentation for the ventral diencephalic area, including the hypothalamus (shown in grey). Furthermore, brainstem segmentation was done for the sampling of the brainstem. Moreover, using dMRI tractography, the DVC-corticolimbic fiber system (DVC-CLFS), shown in white, was extracted in this dataset for the purpose of illustrating the neuroimmune network (image and protocol from Dr. Besteher). For dMRI the following acquisition parameters were used: TE/TR = 80/3140 ms; spatial resolution of 1.5mm isotropic voxel size, 6/8^th^ partial Fourier, multi-band acceleration factor of 4, 35 diffusion weighted gradient directions and 1 b=0 image. Data were acquired for both AP and PA phase encoding and FSL Eddy and topup were used for distortion correction. Tractography: We conducted whole brain tractography using a two-tensor unscented Kalman filter (UKF) method (50, 51), as implemented in the ukftractography^3^ package. After obtaining the pre-processed DWI data, we applied the same UKF parameters for all subjects under study, as follows. Tractography was seeded in all voxels within the brain mask where fractional anisotropy (FA) was greater than 0.1. Tracking stopped where the FA value fell below 0.08 or the normalized mean signal (the sum of the normalized signal across all gradient directions) fell below 0.06. The normalized average signal measure was employed to robustly distinguish between white/gray matter and cerebrospinal fluid (CSF) regions. These seeding and stopping thresholds were set slightly below the default values to enable higher sensitivity for fiber tracking.

Recent neuroimaging studies in COVID-19 have shown the presence of neuroinflammation across several brain areas. More specifically, these studies have corroborated previous findings derived by histopathology in acute COVID-19, in which microglia activation and neuroinflammation have been shown in several brain areas such as the frontal lobes, olfactory bulbs, hippocampus, cerebellum and brainstem. PET studies in particular, have shown alterations in the superior and middle frontal cortex, the anterior, middle and posterior cingulate cortex, the thalamus, hippocampus, cerebellum and brainstem (see e.g., 23–25, 62, 134, 135). Diffuse alterations in the brain, involving the neocortex, thalamus, striatum, hippocampus, cerebellum and brainstem have also been reported recently in a PET study combined with histopathology in macaque monkeys (136). Importantly, these brain areas include also the principal component structures of the neuroimmune network, such as the NTS, DMN, PVN and the dorsal vagal complex (DVC)-corticolimbic connections (**Figure 4**). The diffuse pathological alterations across brain regions such as the frontal lobe, cingulate gyrus, diencephalon and brainstem indicate that autonomic, limbic and cognitive systems should be compromised, which is reflected in the clinical and behavioral phenomenology of acute COVID-19 and also Long COVID. Among the most frequent PASC symptoms that have been reported are fatigue and brain fog characterized by altered cognitive functions, such as attention, memory and executive function in particular (137) as well as vegetative symptoms, including increased temperature, heart rate, and breathing difficulties. The assessment of these symptoms is addressed by using an array of behavioral and clinical tests and batteries. The most frequently used clinical assessment batteries are the Trial Making Test (138) and the Montreal Cognitive Assessment (139) for cognitive evaluation, and the Fatigue Severity Scale (140) for the assessment of fatigue. Finally, to evaluate vegetative symptoms, a checklist of symptoms is used including questions regarding subjective feelings of confusion, drowsiness, cold or heat sensations, nausea as well as assessment of breathing function, sleep and heart rate.

## 3 Conclusions and future directions

The purpose in studying the neuroimmune network using multimodal neuroimaging in acute and post-acute chronic COVID-19 is to detect pathological alterations such as inflammation in that circuitry clinically, i.e., *in vivo*. Most of the anatomical evidence for neuroinflammation in disease states including COVID-19 comes from ex vivo neuropathological studies. Although neuroimaging techniques provide critical insight in localizing and understanding the nature of a brain lesion in many circumstances, brain autopsy followed by histopathological examination still remains the hallmark for understanding disease. Moreover, when anatomopathological investigation focuses on small-sized brain structures such as the NTS, DMN of the vagus and PVN, which are difficult to characterize using routine neuroimaging, the need for autopsy and histopathology is even more evident. In this review we indicate that current neuroimaging of the principal structural components of the neuroimmune network, i.e., the NTS, DMN of the vagus, PVN and the principal fiber pathways connecting these three structures such as the MFB and DLF, combined with *ad hoc* clinical autopsies and histopathological analyses can play a key role in gaining important insight regarding the neuroanatomical mechanisms related to the effects of SARS-CoV-2 infection in the neuroimmune brain circuitry. There remain several unanswered questions regarding the anatomy and histopathology of the neuroimmune network and the pathophysiology of acute COVID-19 and Long COVID. Nevertheless, neuroimaging has shown the potential to contribute significantly in this endeavor, given the clear advantages it offers in clinical research. Namely, neuroimaging enables us to explore the entire brain and not just a subset of structures and locations as is done with routine histopathology. To achieve a holistic and also detailed picture of the histopathological process we need to perform serial sectioning of the entire brain, which, given the cost and time requirements, is a practice that has been abandoned since the 1980s (Dr. Charles Miller Fisher, personal communication). Furthermore, using neuroimaging we are able to monitor disease progression as well as treatment efficacy. This is relevant in understanding whether and how chronic neuroinflammation may lead to neurodegeneration, a hypothesis advanced recently in Long COVID (e.g., 72) and other disorders (e.g., 29, 72, 115). Moreover, we can perform neuroanatomical-clinical correlations. That said, we need to validate neuroimaging findings with histopathology, which remains the hallmark and gold standard for understanding disease. As we develop improved imaging techniques and protocols for studying neuroinflammation (e.g., 141) or other processes, we need to consider seriously the validation aspect of neuroimaging using the traditional radiological-anatomical, radiological-histological and radiological-histopathological correlation approaches. Integrating radiology with histopathology will add validity to neuroimaging, which will become an undisputable tool for translation of anatomical and histopathological information in the clinical domain and, thus at the bedside, a highly beneficial innovation for patients and public health. For all these reasons, we believe that the use of clinical autopsy followed by histopathological examination of the brain tissue for the purpose of direct comparison and correlation with neuroimaging data of the identical tissue, is highly relevant and its practice should be encouraged and supported. This should be the case also for pathological conditions such as neuroinflammation. Furthermore, it is equally important to keep in mind that in current clinical practice, there are no imaging techniques yet available to reveal brain structure at a scale matching histological-microscopic observation. Thus, a histopathological examination remains necessary to gain a thorough understanding of the disease process. It is important to realize that the capability to identify and assess by neuroimaging a specific brain circuitry such as the neuroimmune network in normal and clinical conditions in the individual subject will empower basic and clinical research in an unprecedented way.

## Author contributions

ZK: Writing – original draft. AC-P: Writing – original draft. JG-M: Writing – original draft. RR: Writing – original draft. PT: Writing – original draft. KH: Writing – original draft. GP: Writing – original draft. YR: Writing – original draft. MK: Writing – original draft. RK: Writing – original draft. CH: Writing – original draft. EY: Writing – original draft. BB: Writing – original draft. SP: Writing – original draft. NM: Writing – original draft.

## References

[1] Andersson U Tracey KJ. Reflex principles of immunological homeostasis. Annu Rev Immunol. (2012) 30:313–35. doi: 10.1146/annurev-immunol-020711-075015 PMC4533843 22224768

[2] Niijima A. Effects of taste stimulation on the efferent activity of the autonomic nerves in the rat. Brain Res Bull. (1991) 26:165–7. doi: 10.1016/0361-9230(91)90203-v 2015513

[3] Watkins LR Goehler LE Relton JK Tartaglia N Silbert L Martin D. Blockade of interleukin-1 induced hyperthermia by subdiaphragmatic vagotomy: evidence for vagal mediation of immune-brain communication. Neurosci Lett. (1995) 183:27–31. doi: 10.1016/0304-3940(94)11105-r 7746479

[4] Niijima A. The afferent discharges from sensors for interleukin 1 beta in the hepatoportal system in the anesthetized rat. J Auton Nerv Syst. (1996) 61(3):287–91. doi: 10.1016/s0165-1838(96)00098-7 8988487

[5] Niijima A Hori T Katafuchi T Ichijo T. The effect of interleukin-1 beta on the efferent activity of the vagus nerve to the thymus. J Auton Nerv Syst. (1995) 54:137–44. doi: 10.1016/0165-1838(95)00003-g 7499725

[6] Hori T Katafuchi T Take S Shimizu N Niijima A. The autonomic nervous system as a communication channel between the brain and the immune system. Neuroimmunomodulation. (1995) 2:203–15. doi: 10.1159/000097198 8963749

[7] Tracey KJ. The inflammatory reflex. Nature. (2002) 420:853–9. doi: 10.1038/nature01321 12490958

[8] Cowan WM Harter DH Kandel ER. The emergence of modern neuroscience: some implications for neurology and psychiatry. Annu Rev Neurosci. (2000) 23:343–91. doi: 10.1146/annurev.neuro.23.1.343 10845068

[9] Quadt L Critchley HD Garfinkel SN. The neurobiology of interoception in health and disease. Ann N Y Acad Sci. (2018) 1428:112–28. doi: 10.1111/nyas.13915 29974959

[10] Nelson T Zhang LX Guo H Nacul L Song X. Brainstem abnormalities in myalgic encephalomyelitis/chronic fatigue syndrome: A scoping review and evaluation of magnetic resonance imaging findings. Front Neurol. (2021) 12:769511. doi: 10.3389/fneur.2021.769511 34975729 PMC8718708

[11] Tate W Walker M Sweetman E Helliwell A Peppercorn K Edgar C. Molecular mechanisms of neuroinflammation in ME/CFS and long COVID to sustain disease and promote relapses. Front Neurol. (2022) 13:877772. doi: 10.3389/fneur.2022.877772 35693009 PMC9174654

[12] Tate WP Walker MOM Peppercorn K Blair ALH Edgar CD. Towards a better understanding of the complexities of myalgic encephalomyelitis/chronic fatigue syndrome and long COVID. Int J Mol Sci. (2023) 24:5124. doi: 10.3390/ijms24065124 36982194 PMC10048882

[13] Mehta P McAuley DF Brown M Sanchez E Tattersall RS Manson JJ. COVID-19: consider cytokine storm syndromes and immunosuppression. Lancet. (2020) 395:1033–4. doi: 10.1016/S0140-6736(20)30628-0 PMC7270045 32192578

[14] Moore JB June CH. Cytokine release syndrome in severe COVID-19. Science. (2020) 368:473–4. doi: 10.1126/science.abb8925 32303591

[15] Wang MY Zhao R Gao LJ Gao XF Wang DP Cao JM. SARS-CoV-2: structure, biology, and structure-based therapeutics development. Front Cell Infect Microbiol. (2020) 10:587269. doi: 10.3389/fcimb.2020.587269 33324574 PMC7723891

[16] Yang H Zeng Q Silverman HA Gunasekaran M George SJ Devarajan A. HMGB1 released from nociceptors mediates inflammation. Proc Natl Acad Sci U S A. (2021) 118:e2102034118. doi: 10.1073/pnas.2102034118 34385304 PMC8379951

[17] Fodor A Tiperciuc B Login C Orasan OH Lazar AL Buchman C. Endothelial dysfunction, inflammation, and oxidative stress in COVID-19-mechanisms and therapeutic targets. Oxid Med Cell Longev. (2021) 2021:8671713. doi: 10.1155/2021/8671713 34457119 PMC8397545

[18] Xu E Xie Y Al-Aly Z. Long-term neurologic outcomes of COVID-19. Nat Med. (2022) 28:2406–15. doi: 10.1038/s41591-022-02001-z PMC9671811 36138154

[19] Gladka MM Maack C. The endothelium as Achilles' heel in COVID-19 patients. Cardiovasc Res. (2020) 116:e195–7. doi: 10.1093/cvr/cvaa327 PMC7717148 33167001

[20] Nägele MP Haubner B Tanner FC Ruschitzka F Flammer AJ. Endothelial dysfunction in COVID-19: Current findings and therapeutic implications. Atherosclerosis. (2020) 314:58–62. doi: 10.1016/j.atherosclerosis.2020.10.014 33161318 PMC7554490

[21] Schnaubelt S Oppenauer J Tihanyi D Mueller M Maldonado-Gonzalez E Zejnilovic S. Arterial stiffness in acute COVID-19 and potential associations with clinical outcome. J Intern Med. (2021) 290:437–43. doi: 10.1111/joim.13275 PMC8013324 33651387

[22] Ambrosino P Calcaterra IL Mosella M Formisano R D'Anna SE Bachetti T. Endothelial dysfunction in COVID-19: A unifying mechanism and a potential therapeutic target. Biomedicines. (2022) 10:812. doi: 10.3390/biomedicines10040812 35453563 PMC9029464

[23] Nakatomi Y Mizuno K Ishii A Wada Y Tanaka M Tazawa S. Neuroinflammation in patients with chronic fatigue syndrome/myalgic encephalomyelitis: an ¹¹C-(R)-PK11195 PET study. J Nucl Med. (2014) 55:945–50. doi: 10.2967/jnumed.113.131045 24665088

[24] VanElzakker MB Brumfield SA Lara Mejia PS. Neuroinflammation and cytokines in myalgic encephalomyelitis/chronic fatigue syndrome (ME/CFS): A critical review of research methods. Front Neurol. (2019) 9:1033. doi: 10.3389/fneur.2018.01033 30687207 PMC6335565

[25] Visser D Verfaillie SCJ Wolters EE Coomans EM Timmers T Tuncel H. Differential associations between neocortical tau pathology and blood flow with cognitive deficits in early-onset vs late-onset Alzheimer's disease. Eur J Nucl Med Mol Imaging. (2022) 49:1951–63. doi: 10.1007/s00259-021-05669-6 PMC9016024 34997294

[26] Wu YC Alexander AL. Hybrid diffusion imaging. Neuroimage. (2007) 36:617–29. doi: 10.1016/j.neuroimage.2007.02.050 PMC2428345 17481920

[27] Syková E Nicholson C. Diffusion in brain extracellular space. Physiol Rev. (2008) 88:1277–340. doi: 10.1152/physrev.00027.2007 PMC2785730 18923183

[28] Jacobs AH Tavitian B INMiND consortium. Noninvasive molecular imaging of neuroinflammation. J Cereb Blood Flow Metab. (2012) 32:1393–415. doi: 10.1038/jcbfm.2012.53 PMC3390799 22549622

[29] Pasternak O Kubicki M Shenton ME. *In vivo* imaging of neuroinflammation in schizophrenia. Schizophr Res. (2016) 173:200–12. doi: 10.1016/j.schres.2015.05.034 PMC4668243 26048294

[30] Sapir T Averch Z Lerman B Bodzin A Fishman Y Maitra R. COVID-19 and the immune response: A multi-phasic approach to the treatment of COVID-19. Int J Mol Sci. (2022) 23:8606. doi: 10.3390/ijms23158606 35955740 PMC9369212

[31] Huang C Wang Y Li X Ren L Zhao J Hu Y. Clinical features of patients infected with 2019 novel coronavirus in Wuhan, China. Lancet. (2020) 395:497–506. doi: 10.1016/S0140-6736(20)30183-5 31986264 PMC7159299

[32] Karki R Sharma BR Tuladhar S Williams EP Zalduondo L Samir P. Synergism of TNF-α and IFN-γ Triggers inflammatory cell death, tissue damage, and mortality in SARS-coV-2 infection and cytokine shock syndromes. Cell. (2021) 184:149–168.e17. doi: 10.1016/j.cell.2020.11.025 33278357 PMC7674074

[33] Triggle CR Bansal D Ding H Islam MM Farag EABA Hadi HA. A comprehensive review of viral characteristics, transmission, pathophysiology, immune response, and management of SARS-CoV-2 and COVID-19 as a basis for controlling the pandemic. Front Immunol. (2021) 12:631139. doi: 10.3389/fimmu.2021.631139 33717166 PMC7952616

[34] Chen LYC Quach TTT. COVID-19 cytokine storm syndrome: a threshold concept. Lancet Microbe. (2021) 2:e49–50. doi: 10.1016/S2666-5247(20)30223-8 PMC7906728 33655230

[35] Xu SW Ilyas I Weng JP. Endothelial dysfunction in COVID-19: an overview of evidence, biomarkers, mechanisms and potential therapies. Acta Pharmacol Sin. (2023) 44:695–709. doi: 10.1038/s41401-022-00998-0 36253560 PMC9574180

[36] Adang EAMC Strous MTA van den Bergh JP Gach D van Kampen VEM van Zeeland REP. Association of heart rate variability with pulmonary function impairment and symptomatology post-COVID-19 hospitalization. Sensors (Basel). (2023) 23:2473. doi: 10.3390/s23052473 36904676 PMC10007596

[37] Soriano JB Murthy S Marshall JC Relan P Diaz JV. WHO Clinical Case Definition Working Group on Post-COVID-19 Condition. A clinical case definition of post-COVID-19 condition by a Delphi consensus. Lancet Infect Dis. (2022) 22:e102–7. doi: 10.1016/S1473-3099(21)00703-9 PMC8691845 34951953

[38] Hussain M Khurram Syed S Fatima M Shaukat S Saadullah M Alqahtani AM. Acute respiratory distress syndrome and COVID-19: A literature review. J Inflammation Res. (2021) 14:7225–42. doi: 10.2147/JIR.S334043 PMC8710428 34992415

[39] Filbin MR. Insights into endotheliopathy in COVID-19. Am J Respir Crit Care Med. (2022) 206:926–8. doi: 10.1164/rccm.202207-1258ED PMC9801991 35819867

[40] Six I Guillaume N Jacob V Mentaverri R Kamel S Boullier A. The endothelium and COVID-19: an increasingly clear link brief title: endotheliopathy in COVID-19. Int J Mol Sci. (2022) 23:6196. doi: 10.3390/ijms23116196 35682871 PMC9181280

[41] Fotuhi M Mian A Meysami S Raji CA. Neurobiology of COVID-19. J Alzheimers Dis. (2020) 76:3–19. doi: 10.3233/JAD-200581 32538857 PMC7660990

[42] Taquet M Dercon Q Luciano S Geddes JR Husain M Harrison PJ. Incidence, co-occurrence, and evolution of long-COVID features: A 6-month retrospective cohort study of 273,618 survivors of COVID-19. PloS Med. (2021) 18:e1003773. doi: 10.1371/journal.pmed.1003773 34582441 PMC8478214

[43] Vanderheiden A Klein RS. Neuroinflammation and COVID-19. Curr Opin Neurobiol. (2022) 76:102608. doi: 10.1016/j.conb.2022.102608 35863101 PMC9239981

[44] Matschke J Lütgehetmann M Hagel C Sperhake JP Schröder AS Edler C. Neuropathology of patients with COVID-19 in Germany: a post-mortem case series. Lancet Neurol. (2020) 19:919–29. doi: 10.1016/S1474-4422(20)30308-2 PMC7535629 33031735

[45] Schurink B Roos E Radonic T Barbe E Bouman CSC de Boer HH. Viral presence and immunopathology in patients with lethal COVID-19: a prospective autopsy cohort study. Lancet Microbe. (2020) 1:e290–9. doi: 10.1016/S2666-5247(20)30144-0 PMC7518879 33015653

[46] Solomon IH Normandin E Bhattacharyya S Mukerji SS Keller K Ali AS. Neuropathological features of covid-19. N Engl J Med. (2020) 383:989–92. doi: 10.1056/NEJMc2019373 PMC7304421 32530583

[47] DaSilva AF Becerra L Makris N Strassman AM Gonzalez RG Geatrakis N. Somatotopic activation in the human trigeminal pain pathway. J Neurosci. (2002) 22:8183–92. doi: 10.1523/JNEUROSCI.22-18-08183.2002 PMC6758094 12223572

[48] Makris N Hodge SM Breiter HC McInerney SC Haselgrove C Kennedy DN. MRI based topographic parcellation of human brainstem with systematics of corticopontine connectivity, in: 9th Annual Meeting of the Organization for Human Brain Mapping (OHBM 2003), New York, NY.

[49] Yang JC Papadimitriou G Eckbo R Yeterian EH Liang L Dougherty DD. Multi-tensor investigation of orbitofrontal cortex tracts affected in subcaudate tractotomy. Brain Imaging Behav. (2015) 9:342–52. doi: 10.1007/s11682-014-9314-z PMC4320992 25103312

[50] Malcolm JG Shenton ME Rathi Y. Filtered multitensor tractography. IEEE Trans Med Imaging. (2010) 29:1664–75. doi: 10.1109/TMI.2010.2048121 PMC3045040 20805043

[51] Reddy CP Rathi Y. Joint multi-fiber NODDI parameter estimation and tractography using the unscented information filter. Front Neurosci. (2016) 10:1665. doi: 10.3389/fnins.2016.00166 PMC4837399 27147956

[52] Rivas-Grajales AM Sawyer KS Karmacharya S Papadimitriou G Camprodon JA Harris GJ. Sexually dimorphic structural abnormalities in major connections of the medial forebrain bundle in alcoholism. NeuroImage Clin. (2018) 19:98–105. doi: 10.1016/j.nicl.2018.03.025 30035007 PMC6051309

[53] Puelles VG Lütgehetmann M Lindenmeyer MT Sperhake JP Wong MN Allweiss L. Multiorgan and renal tropism of SARS-CoV-2. N Engl J Med. (2020) 383:590–2. doi: 10.1056/NEJMc2011400 PMC7240771 32402155

[54] Zamani R Pouremamali R Rezaei N. Central neuroinflammation in Covid-19: a systematic review of 182 cases with encephalitis, acute disseminated encephalomyelitis, and necrotizing encephalopathies. Rev Neurosci. (2021) 33:397–412. doi: 10.1515/revneuro-2021-0082 34536341

[55] De Felice FG Tovar-Moll F Moll J Munoz DP Ferreira ST. Severe acute respiratory syndrome coronavirus 2 (SARS-CoV-2) and the central nervous system. Trends Neurosci. (2020) 43:355–7. doi: 10.1016/j.tins.2020.04.004 PMC7172664 32359765

[56] Platt MP Bolding KA Wayne CR Chaudhry S Cutforth T Franks KM. Th17 lymphocytes drive vascular and neuronal deficits in a mouse model of postinfectious autoimmune encephalitis. Proc Natl Acad Sci U S A. (2020) 117:6708–16. doi: 10.1073/pnas.1911097117 PMC7104239 32161123

[57] Davis HE McCorkell L Vogel JM Topol EJ. Long COVID: major findings, mechanisms and recommendations. Nat Rev Microbiol. (2023) 21:133–46. doi: 10.1038/s41579-022-00846-2 PMC9839201 36639608

[58] Ballering AV van Zon SKR Olde Hartman TC Rosmalen JGM. Lifelines Corona Research Initiative. Persistence of somatic symptoms after COVID-19 in the Netherlands: an observational cohort study. Lancet. (2022) 400:452–61. doi: 10.1016/S0140-6736(22)01214-4 PMC9352274 35934007

[59] Kedor C Freitag H Meyer-Arndt L Wittke K Hanitsch LG Zoller T. Author Correction: A prospective observational study of post-COVID-19 chronic fatigue syndrome following the first pandemic wave in Germany and biomarkers associated with symptom severity. Nat Commun. (2022) 13:6009. doi: 10.1038/s41467-022-33784-x. Erratum for: Nat Commun. 2022 Aug 30;13(1):5104. 36042189 PMC9426365

[60] Larsen NW Stiles LE Shaik R Schneider L Muppidi S Tsui CT. Characterization of autonomic symptom burden in long COVID: A global survey of 2,314 adults. Front Neurol. (2022) 13:1012668. doi: 10.3389/fneur.2022.1012668 36353127 PMC9639503

[61] Cairns R Hotopf M. A systematic review describing the prognosis of chronic fatigue syndrome. Occup Med (Lond). (2005) 55:20–31. doi: 10.1093/occmed/kqi013 15699087

[62] Rossi S Prodi E Morese R Paone G Ruberto T Sacco L. Persistent 18F-FDG brain PET fronto-temporal hypometabolism and cognitive symptoms two years after SARS-CoV-2 infection: A case report. Neurol Int. (2023) 15:908–16. doi: 10.3390/neurolint15030058 PMC10443341 37606391

[63] Leng A Shah M Ahmad SA Premraj L Wildi K Li Bassi G. Pathogenesis underlying neurological manifestations of long COVID syndrome and potential therapeutics. Cells. (2023) 12:816. doi: 10.3390/cells12050816 36899952 PMC10001044

[64] Monje M Iwasaki A. The neurobiology of long COVID. Neuron. (2022) 110:3484–96. doi: 10.1016/j.neuron.2022.10.006 PMC9537254 36288726

[65] Obermeier B Daneman R Ransohoff RM. Development, maintenance and disruption of the blood-brain barrier. Nat Med. (2013) 19:1584–96. doi: 10.1038/nm.3407 PMC4080800 24309662

[66] Zhao Z Nelson AR Betsholtz C Zlokovic BV. Establishment and dysfunction of the blood-brain barrier. Cell. (2015) 163:1064–78. doi: 10.1016/j.cell.2015.10.067 PMC4655822 26590417

[67] Alexopoulos H Magira E Bitzogli K Kafasi N Vlachoyiannopoulos P Tzioufas A. Anti-SARS-CoV-2 antibodies in the CSF, blood-brain barrier dysfunction, and neurological outcome: Studies in 8 stuporous and comatose patients. Neurol Neuroimmunol Neuroinflamm. (2020) 7:e893. doi: 10.1212/NXI.0000000000000893 32978291 PMC7577546

[68] Bodro M Compta Y Llansó L Esteller D Doncel-Moriano A Mesa A. “Hospital Clínic Infecto-COVID-19” and “Hospital Clínic Neuro-COVID-19” groups. Increased CSF levels of IL-1β, IL-6, and ACE in SARS-CoV-2-associated encephalitis. Neurol Neuroimmunol Neuroinflamm. (2020) 7:e821. doi: 10.1212/NXI.0000000000000821 32611761 PMC7357418

[69] Lengfeld JE Lutz SE Smith JR Diaconu C Scott C Kofman SB. Endothelial Wnt/β-catenin signaling reduces immune cell infiltration in multiple sclerosis. Proc Natl Acad Sci U S A. (2017) 114:E1168–77. doi: 10.1073/pnas.1609905114 PMC5320985 28137846

[70] Harris WJ Asselin MC Hinz R Parkes LM Allan S Schiessl I. *In vivo* methods for imaging blood-brain barrier function and dysfunction. Eur J Nucl Med Mol Imaging. (2023) 50:1051–83. doi: 10.1007/s00259-022-05997-1 PMC9931809 36437425

[71] Tietz SM Engelhardt B. Visualizing impairment of the endothelial and glial barriers of the neurovascular unit during experimental autoimmune encephalomyelitis *in vivo* . J Vis Exp. (2019) 145. doi: 10.3791/59249 30985749

[72] McAlpine LS Fesharaki-Zadeh A Spudich S. Coronavirus disease 2019 and neurodegenerative disease: what will the future bring? Curr Opin Psychiatry. (2021) 34:177–85. doi: 10.1097/YCO.0000000000000688 PMC7924921 33395100

[73] Rudnicka-Drożak E Drożak P Mizerski G Zaborowski T Ślusarska B Nowicki G. Links between COVID-19 and alzheimer's disease-what do we already know? Int J Environ Res Public Health. (2023) 20:2146. doi: 10.3390/ijerph20032146 36767513 PMC9915236

[74] Kasparian K Graykowski D Cudaback E. Commentary: APOE e4 genotype predicts severe COVID-19 in the UK biobank community cohort. Front Immunol. (2020) 11:1939. doi: 10.3389/fimmu.2020.01939 33042114 PMC7522160

[75] Kuo CL Pilling LC Atkins JL Masoli JAH Delgado J Kuchel GA. ApoE e4e4 genotype and mortality with COVID-19 in UK biobank. J Gerontol A Biol Sci Med Sci. (2020) 75:1801–3. doi: 10.1093/gerona/glaa169 PMC7337688 32623451

[76] Verghese PB Castellano JM Holtzman DM. Apolipoprotein E in Alzheimer's disease and other neurological disorders. Lancet Neurol. (2011) 10:241–52. doi: 10.1016/S1474-4422(10)70325-2 PMC3132088 21349439

[77] Marwaha B. Role of Tau protein in long COVID and potential therapeutic targets. Front Cell Infect Microbiol. (2023) 13:1280600. doi: 10.3389/fcimb.2023.1280600 37953801 PMC10634420

[78] Ramani A Müller L Ostermann PN Gabriel E Abida-Islam P Müller-Schiffmann A. SARS-CoV-2 targets neurons of 3D human brain organoids. EMBO J. (2020) 39:e106230. doi: 10.15252/embj.2020106230 32876341 PMC7560208

[79] de Calignon A Polydoro M Suárez-Calvet M William C Adamowicz DH Kopeikina KJ. Propagation of tau pathology in a model of early Alzheimer's disease. Neuron. (2012) 73:685–97. doi: 10.1016/j.neuron.2011.11.033. Erratum in: Neuron. 2012 Oct 18;76(2):461. PMC3292759 22365544

[80] Wang C Fan L Khawaja RR Liu B Zhan L Kodama L. Microglial NF-κB drives tau spreading and toxicity in a mouse model of tauopathy. Nat Commun. (2022) 13:1969. doi: 10.1038/s41467-022-29552-6 35413950 PMC9005658

[81] Goetzl EJ Mustapic M Kapogiannis D Eitan E Lobach IV Goetzl L. Cargo proteins of plasma astrocyte-derived exosomes in Alzheimer's disease. FASEB J. (2016) 30:3853–9. doi: 10.1096/fj.201600756R PMC5067254 27511944

[82] Wang L Davis PB Volkow ND Berger NA Kaelber DC Xu R. Association of COVID-19 with new-onset alzheimer's disease. J Alzheimers Dis. (2022) 89:411–4. doi: 10.3233/JAD-220717 PMC10361652 35912749

[83] Pradeu T. Philosophy of immunology (Elements in the philosophy of biology). Cambridge: Cambridge University Press (2020). doi: 10.1017/9781108616706

[84] Borovikova LV Ivanova S Zhang M Yang H Botchkina GI Watkins LR. Vagus nerve stimulation attenuates the systemic inflammatory response to endotoxin. Nature. (2000) 405:458–62. doi: 10.1038/35013070 10839541

[85] Borovikova LV Ivanova S Nardi D Zhang M Yang H Ombrellino M. Role of vagus nerve signaling in CNI-1493-mediated suppression of acute inflammation. Auton Neurosci. (2000) 85:141–7. doi: 10.1016/S1566-0702(00)00233-2 11189021

[86] Blalock JE. The immune system as a sensory organ. J Immunol. (1984) 132:1067–70. 6363533

[87] Blalock JE. The syntax of immune-neuroendocrine communication. Immunol Today. (1994) 15:504–11. doi: 10.1016/0167-5699(94)90205-4 7802919

[88] Rosas-Ballina M Tracey KJ. The neurology of the immune system: neural reflexes regulate immunity. Neuron. (2009) 64:28–32. doi: 10.1016/j.neuron.2009.09.039 19840545 PMC4533851

[89] Jänig W. Integrative action of the autonomic nervous system: neurobiology of homeostasis. Cambridge: Cambridge University Press (2006). doi: 10.1017/CBO9780511541667

[90] Nieuwenhuys R. Chemoarchitecture of the brain. Heidelberg: Springer-Verlag Berlin (1985).

[91] Chrousos GP. The stress response and immune function: clinical implications. The 1999 Novera H. Spector Lecture. Ann N Y Acad Sci. (2000) 917:38–67. doi: 10.1111/j.1749-6632.2000.tb05371.x 11268364

[92] Molina PE. Noradrenergic inhibition of TNF upregulation in hemorrhagic shock. Neuroimmunomodulation. (2001) 9:125–33. doi: 10.1159/000049016 11752885

[93] Molina PE Bagby GJ Stahls P. Hemorrhage alters neuroendocrine, hemodynamic, and compartment-specific TNF responses to LPS. Shock. (2001) 16:459–65. doi: 10.1097/00024382-200116060-00010 11770045

[94] Mackay A Tate WP. A compromised paraventricular nucleus within a dysfunctional hypothalamus: A novel neuroinflammatory paradigm for ME/CFS. Int J Immunopathology Pharmacol. (2018) 32. doi: 10.1177/2058738418812342

[95] Mackay AA. Paradigm for post-covid-19 fatigue syndrome analogous to ME/CFS. Front Neurol. (2021) 12:701419. doi: 10.3389/fneur.2021.701419 34408721 PMC8365156

[96] Castañeyra-Perdomo A Meyer G Heylings DJ. Early development of the human area postrema and subfornical organ. Anat Rec. (1992) 232:612–9. doi: 10.1002/ar.1092320416 1554110

[97] Ganong WF. Circumventricular organs: definition and role in the regulation of endocrine and autonomic function. Clin Exp Pharmacol Physiol. (2000) 27:422–7. doi: 10.1046/j.1440-1681.2000.03259.x 10831247

[98] Goren O Adorján I Kálmán M. Heterogeneous occurrence of aquaporin-4 in the ependyma and in the circumventricular organs in rat and chicken. Anat Embryol (Berl). (2006) 211:155–72. doi: 10.1007/s00429-005-0067-8 16416308

[99] Roemer SF Parisi JE Lennon VA Benarroch EE Lassmann H Bruck W. Pattern-specific loss of aquaporin-4 immunoreactivity distinguishes neuromyelitis optica from multiple sclerosis. Brain. (2007) 130:1194–205. doi: 10.1093/brain/awl371 17282996

[100] Gutman MB Ciriello J Mogenson GJ. Effects of plasma angiotensin II and hypernatremia on subfornical organ neurons. Am J Physiol. (1988) 254:R746–54. doi: 10.1152/ajpregu.1988.254.5.R746 3364604

[101] Anderson JW Smith PM Ferguson AV. Subfornical organ neurons projecting to paraventricular nucleus: whole-cell properties. Brain Res. (2001) 921:78–85. doi: 10.1016/s0006-8993(01)03093-1 11720713

[102] Nieuwenhuys R Voogd J Huijzen C. The human central nervous system. Heidelberg: Springer-Verlag Berlin (1988).

[103] Nolte J. The human brain: an introduction to its functional anatomy. Nolte; Philadelphia, PA: Mosby Elsevier (2009).

[104] Shanks J Ramchandra R. Angiotensin II and the cardiac parasympathetic nervous system in hypertension. Int J Mol Sci. (2021) 22:12305. doi: 10.3390/ijms222212305 34830184 PMC8624735

[105] Veerasingham SJ Raizada MK. Brain renin-angiotensin system dysfunction in hypertension: recent advances and perspectives. Br J Pharmacol. (2003) 139:191–202. doi: 10.1038/sj.bjp.0705262 12770924 PMC1573858

[106] Haspula D Clark MA. Molecular basis of the brain renin angiotensin system in cardiovascular and neurologic disorders: uncovering a key role for the astroglial angiotensin type 1 receptor AT1R. J Pharmacol Exp Ther. (2018) 366:251–64. doi: 10.1124/jpet.118.248831 29752427

[107] Miller AJ Arnold AC. The renin-angiotensin system in cardiovascular autonomic control: recent developments and clinical implications. Clin Auton Res. (2019) 29:231–43. doi: 10.1007/s10286-018-0572-5 PMC6461499 30413906

[108] Nehme A Zouein FA Zayeri ZD Zibara K. An update on the tissue renin angiotensin system and its role in physiology and pathology. J Cardiovasc Dev Dis. (2019) 6:14. doi: 10.3390/jcdd6020014 30934934 PMC6617132

[109] Schwartz M. Macrophages and microglia in central nervous system injury: are they helpful or harmful? J Cereb Blood Flow Metab. (2003) 23:385–94. doi: 10.1097/01.WCB.0000061881.75234.5E 12679714

[110] Versijpt J Debruyne JC Van Laere KJ De Vos F Keppens J Strijckmans K. Microglial imaging with positron emission tomography and atrophy measurements with magnetic resonance imaging in multiple sclerosis: a correlative study. Mult Scler. (2005) 11:127–34. doi: 10.1191/1352458505ms1140oa 15794383

[111] Deng W. Neurobiology of injury to the developing brain. Nat Rev Neurol. (2010) 6:328–36. doi: 10.1038/nrneurol.2010.53 20479779

[112] Bigler ED. Neuroinflammation and the dynamic lesion in traumatic brain injury. Brain. (2013) 136:9–11. doi: 10.1093/brain/aws342 23365089

[113] Chew LJ Fusar-Poli P Schmitz T. Oligodendroglial alterations and the role of microglia in white matter injury: relevance to schizophrenia. Dev Neurosci. (2013) 35:102–29. doi: 10.1159/000346157 PMC4531048 23446060

[114] Frodl T Amico F. Is there an association between peripheral immune markers and structural/functional neuroimaging findings? Prog Neuropsychopharmacol Biol Psychiatry. (2014) 48:295–303. doi: 10.1016/j.pnpbp.2012.12.013 23313563

[115] Feigenson KA Kusnecov AW Silverstein SM. Inflammation and the two-hit hypothesis of schizophrenia. Neurosci Biobehav Rev. (2014) 38:72–93. doi: 10.1016/j.neubiorev.2013.11.006 24247023 PMC3896922

[116] Fuchs VR Sox HC Jr. Physicians' views of the relative importance of thirty medical innovations. Health Aff (Millwood). (2001) 20:30–42. doi: 10.1377/hlthaff.20.5.30 11558715

[117] Kopp N. How technologies of imaging are shaping clinical research and practice in neurology. Med Stud. (2009) 1:315–28. doi: 10.1007/s12376-010-0037-1

[118] Kannan S Balakrishnan B Muzik O Romero R Chugani D. Positron emission tomography imaging of neuroinflammation. J Child Neurol. (2009) 24:1190–9. doi: 10.1177/0883073809338063 PMC3840908 19745091

[119] Venneti S Lopresti BJ Wiley CA. Molecular imaging of microglia/macrophages in the brain. Glia. (2013) 61:10–23. doi: 10.1002/glia.22357 22615180 PMC3580157

[120] Rupprecht R Papadopoulos V Rammes G Baghai TC Fan J Akula N. Translocator protein (18 kDa) (TSPO) as a therapeutic target for neurological and psychiatric disorders. Nat Rev Drug Discovery. (2010) 9:971–88. doi: 10.1038/nrd3295 21119734

[121] Filipek PA Richelme C Kennedy DN Caviness VS Jr. The young adult human brain: an MRI-based morphometric analysis. Cereb Cortex. (1994) 4:344–60. doi: 10.1093/cercor/4.4.344 7950308

[122] Caviness VS Jr Lange NT Makris N Herbert MR Kennedy DN. MRI-based brain volumetrics: emergence of a developmental brain science. Brain Dev. (1999) 21:289–95. doi: 10.1016/s0387-7604(99)00022-4 10413014

[123] Fischl B Salat DH Busa E Albert M Dieterich M Haselgrove C. Whole brain segmentation: automated labeling of neuroanatomical structures in the human brain. Neuron. (2002) 33:341–55. doi: 10.1016/s0896-6273(02)00569-x 11832223

[124] Barnes D McDonald WI Johnson G Tofts PS Landon DN. Quantitative nuclear magnetic resonance imaging: characterisation of experimental cerebral oedema. J Neurol Neurosurg Psychiatry. (1987) 50:125–33. doi: 10.1136/jnnp.50.2.125 PMC1031482 3572428

[125] Claudio L Kress Y Factor J Brosnan CF. Mechanisms of edema formation in experimental autoimmune encephalomyelitis. contribution inflammatory Cells Am J Pathol. (1990) 137:1033–45. PMC1877669 2240157

[126] Stamatovic SM Keep RF Andjelkovic AV. Brain endothelial cell-cell junctions: how to "open" the blood brain barrier. Curr Neuropharmacol. (2008) 6:179–92. doi: 10.2174/157015908785777210 PMC2687937 19506719

[127] Castillo M Mukherji SK. Clinical applications of FLAIR, HASTE, and magnetization transfer in neuroimaging. Semin Ultrasound CT MR. (2000) 21:417–27. doi: 10.1016/s0887-2171(00)90034-9 11138631

[128] Assaf Y Pasternak O. Diffusion tensor imaging (DTI)-based white matter mapping in brain research: a review. J Mol Neurosci. (2008) 34:51–61. doi: 10.1007/s12031-007-0029-0 18157658

[129] Pasternak O Sochen N Gur Y Intrator N Assaf Y. Free water elimination and mapping from diffusion MRI. Magn Reson Med. (2009) 62:717–30. doi: 10.1002/mrm.22055 19623619

[130] Rigotti DJ Inglese M Gonen O. Whole-brain N-acetylaspartate as a surrogate marker of neuronal damage in diffuse neurologic disorders. AJNR Am J Neuroradiol. (2007) 28:1843–9. doi: 10.3174/ajnr.A0774 PMC8134227 17921226

[131] Chang L Munsaka SM Kraft-Terry S Ernst T. Magnetic resonance spectroscopy to assess neuroinflammation and neuropathic pain. J Neuroimmune Pharmacol. (2013) 8:576–93. doi: 10.1007/s11481-013-9460-x PMC3698315 23666436

[132] Oz G Alger JR Barker PB Bartha R Bizzi A Boesch C. Clinical proton MR spectroscopy in central nervous system disorders. Radiology. (2014) 270:658–79. doi: 10.1148/radiol.13130531 PMC4263653 24568703

[133] Breiter HC Gasic GP Makris N. Imaging the neural systems for motivated behavior and their dysfunction in neuropsychiatric illness. In: Deisboeck TS Kresh JY, editors. Complex systems science in biomedicine. Springer, Boston, MA (2006). doi: 10.1007/978-0-387-33532-2_33

[134] Sandiego CM Gallezot JD Pittman B Nabulsi N Lim K Lin SF. Imaging robust microglial activation after lipopolysaccharide administration in humans with PET. Proc Natl Acad Sci U S A. (2015) 112:12468–73. doi: 10.1073/pnas.1511003112 PMC4603509 26385967

[135] Sollini M Morbelli S Ciccarelli M Cecconi M Aghemo A Morelli P. Long COVID hallmarks on [18F]FDG-PET/CT: a case-control study. Eur J Nucl Med Mol Imaging. (2021) 48:3187–97. doi: 10.1007/s00259-021-05294-3 PMC7937050 33677642

[136] Nieuwland JM Nutma E Philippens IHCHM Böszörményi KP Remarque EJ Bakker J. Longitudinal positron emission tomography and postmortem analysis reveals widespread neuroinflammation in SARS-CoV-2 infected rhesus macaques. J Neuroinflammation. (2023) 20:179. doi: 10.1186/s12974-023-02857-z 37516868 PMC10387202

[137] Thaweethai T Jolley SE Karlson EW Levitan EB Levy B McComsey GA. Development of a definition of postacute sequelae of SARS-CoV-2 infection. JAMA. (2023) 329:1934–46. doi: 10.1001/jama.2023.8823 PMC10214179 37278994

[138] Bowie CR Harvey PD. Administration and interpretation of the trail making test. Nat Protoc. (2006) 1:2277–81. doi: 10.1038/nprot.2006.390 17406468

[139] Nasreddine ZS Phillips NA Bédirian V Charbonneau S Whitehead V Collin I. The Montreal Cognitive Assessment, MoCA: a brief screening tool for mild cognitive impairment. J Am Geriatr Soc. (2005) 53:695–9. doi: 10.1111/j.1532-5415.2005.53221.x. Erratum in: J Am Geriatr Soc. 2019 Sep;67(9):1991. 15817019

[140] Siciliano M Chiorri C De Micco R Russo A Tedeschi G Trojano L. Fatigue in Parkinson's disease: Italian validation of the Parkinson Fatigue Scale and the Fatigue Severity Scale using a Rasch analysis approach. Parkinsonism Relat Disord. (2019) 65:105–10. doi: 10.1016/j.parkreldis.2019.05.028 31147224

[141] Castañeyra-Perdomo A González-Mora JL Carmona-Calero EM Makris N Carrasco-Juan JL. An opinion and narrative review on the clinical relevance of imaging the circumventricular brain organs and performing their anatomical and histopathological examination in acute and post-acute COVID-19. Am J Forensic Med Pathol. In Press. 10.1097/PAF.0000000000000939 PMC11479582 38739896

